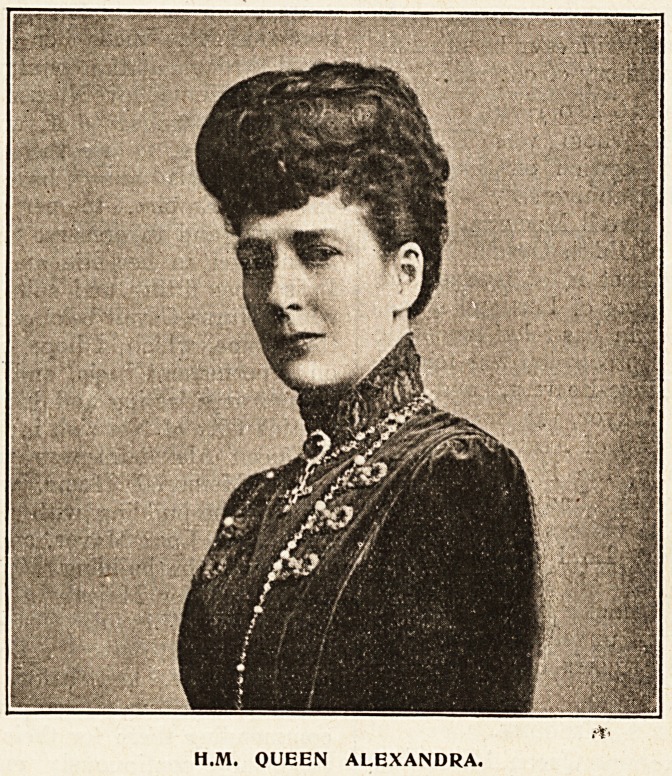# Four Ceremonies: The Cavell Statue, Her Memorial, King Edward VII. Ward, and the Bishop's Palace

**Published:** 1918-10-19

**Authors:** 


### QUEEN ALEXANDRA VISITS NORFOLK'S CAPITAL.

### Four Ceremonies: The Cavell Statue, Her Memorial, King Edward VII. Ward, and the Bishop's Palace.

RESIDENTS in the City of Norwich have for many years taken the deepest interest in nurses and nursing. It will interest readers of THE HOSPITAL to know that the late Louisa Warnes, of beloved memory, was the leading spirit who, in conjunction with Doctor and Miss Gibson, founded the Norfolk and Norwich Staff of Nurses, originally known as the Bethel Street Nurses, so long ago as the year 1866. At that period in East Anglia trained nurses were unknown, but Louisa Warnes foresaw the need, and attracted many good women willing to be trained as nurses at the hospitals. They constituted the first staff of trained nurses for the sick and poor in Norwich and its neighbourhood. Nurses never had a truer or wiser friend than Louisa Warnes. The last day of her active life she spent at Surrey Street, and with those of the nursing staff who were present in the Home. These nurses were in no way connected with the Norfolk and Norwich Hospital, but were engaged in private work among the well-to-do classes until 1903, when the committee started the Norwich District Nurses' Home, which, through the energy of Dr. Taylor, its assistant secretary, was affiliated to the Queen's Jubilee Institute in February 1906. In the following June Miss Arnold, the present superintendent, was appointed to take charge of it, thus becoming the first Queen's superintendent in the County of Norfolk, as this was the first Queen's Home within its boundaries.

At first the Home had only two nurses for district work, but the last annual report shows that the number of cases during the year has been 952, and the number of visits made by the nursing staff 21,015. The accommodation of the Home has been considerably extended, and after the war, when the two shops adjoining it have been added, there will be room for sixteen nurses, the present staff consisting of eight. Miss Arnold is anxious to secure close co-operation between the nursing and health organisations of the City of Norwich. By this arrangement a desirable re-arrangement would be made, whereby the health visitors employed in Norwich could be brought under the roof of the Cavell Memorial Home. Miss Arnold is commendably ambitious that the first result of this Memorial should secure the health visiting, together with tuberculosis and school nursing, all carried out from the Cavell Home. The Home requires £500 to be subscribed to complete the £2,700 needed, towards which the Lord Mayor's Appeal has already brought in £2,200.

H.M. QUEEN ALEXANDRA.

HER MAJESTY QUEEN ALEXANDRA.

Queen Alexandra, best beloved of women, worshipped as she is by the people of Great Britain her adopted country, foremost in active and continuous work for the sailors, soldiers, and airmen, and the sick and injured everywhere, must have regarded her visit to Norwich on Saturday last as a Red-Letter Day in the Royal calendar. For some fifty years the County of Norfolk has been blessed by Queen Alexandra's residence, and the knowledge that Sandringham is regarded by her as the Home of her affections, to which she is greatly attached and where she has usually resided, though disturbed since the war by the brutal Germans deliberately sending squadrons of aeroplanes, without, however, succeeding in finding the object of their search. Brave woman that she is, and fond of her home, her heart has been with Sandringham, where she has so many ties, and where she takes a great personal interest in the lives of the people and all that can minister to their happiness and prosperity. It has become admittedly recognised with increasing conviction that Almighty God has been in this war from the commencement. Having regard to its present condition of progress, due to the splendid

### Queen Alexandra Visits Norfolk's Capital—*(continued)*.

courage and devotion of the men of all ranks in the Navies, Armies, and Air Forces, who have fought with such wonderful tenacity and resolve, to-day is full of promise for the future life and blessedness of the people of the British Empire, of our brothers of the United States, in the union which now cements our hearts and theirs, and of the empires and countries which have joined hands with us to bring freedom to and enforce and preserve the rights of the smaller nations, by the victory of arms and lasting peace, which they have united, with solemn purpose to achieve. King George and Queen Mary have set us all a noble example of untiring energy and personal devotion, by service from the outbreak of the war. Every member of the Royal Family has devoted his or her energies to following Their Majesties' example, amongst whom Queen Alexandra's devoted self-denial and continuous efforts will ever be remembered and treasured in the history of our race.

### The Cavell Memorial and Home.

Queen Alexandra, with whom was H.R.H. Princess Victoria, visited Norwich on Saturday, the 12th instant, the third anniversary of Miss Cavell's death, to open the Cavell Memorial Home and unveil a bronze bust of Edith Cavell, Nurse, Patriot, and Martyr. The bust is of bronze, the pedestal on which it rests being of Portland stone, on which is carved a soldier in bas-relief tendering a wreath of immortelles. A procession was formed consisting of the Civic Mace-Bearers, and the Sword-Bearer, the Deputy Mayor, the Sheriff, the Town Clerk, the Lord Bishop of Norwich, Queen Alexandra, Princess Victoria, the Hon. Charlotte Knollys, the Lady Mayoress, Lady Dartmouth, and Lady Coke. The Royal visitors were received by Colonel Lord Leicester, Lord Lieutenant of Norfolk, the Lord Mayor of Norwich, and other civic representatives. Among the dense crowd of people who witnessed the unveiling were hundreds of wounded soldiers, squads of nurses, and several ambulance units.

### The Lord Mayor's Welcome.

The Lord Mayor, in welcoming Queen Alexandra, said:—

Your Majesty will deem it fitting that in the chief city of Edith Cavell's native county, the home of Mrs. Cavell too, till her recent decease, some permanent memorial should be raised to this Norfolk heroine of whom we are so justifiably proud. This city is already famous as the birthplace of Elizabeth Fry, and we desire to perpetuate the memory of this other noble woman, whose fame is also world-wide. In the splendid work which our nurses are doing, and in the knowledge that kind words and kind deeds can never die, we find reason to hope that the memorial to our martyred heroine will be lasting, for it depends not upon material things, but has for its foundation spiritual realities that are eternal. Edith Cavell rests from her labours and her works do follow her. This is the third anniversary of her death, bringing with it the promise of a lasting and righteous peace, and the hope that the cause for which Nurse Cavell gave her life is about to triumph.

### Nurse Cavell's Statue.

Queen Alexandra then unveiled the bust, which excited great interest and approval.

### Queen Alexandra's Speech.

"I thank you, my Lord Mayor, for the welcome you have given me, and for the kindly expressions you have used with reference to my beloved husband, King Edward (who was so greatly attached to the County of Norfolk), and to myself. It has given me sincere pleasure to visit your ancient and historic City of Norwich to-day, not only because it is the capital of the county in which I live for a great part of the year, and which is endeared to me by the happiest and most tender associations, but because the occasion of unveiling this statue, and opening the Nurses' Home, has given me the opportunity of testifying my admiration and respect for the memory of a brave woman, Nurse Edith Cavell, who met a martyr's fate with a calm courage, an intrepid faith, and a spiritual resignation that have made her name honoured and revered throughout the country and the Empire.

"No Home for Nurses could have worthier memories attached to it, and I should like, if it were possible, to see these Homes established as some, I am glad to say, have already been, throughout the Empire, to perpetuate Nurse Cavell's memory, and to preserve the traditions which she maintained in her life and upheld by her death. It is most fitting and suitable that the county to which Nurse Cavell belonged should have instituted this Home, which, I hope, may now be established on a permanent basis, and may remain, with the statue, as a lasting and historic memorial, erected by this City of Norwich in her honour."

Queen Alexandra was then conducted to the Cavell Memorial Home, where she unlocked the door of the building with a gold key presented to her by the Lord Mayor, and entering it proceeded to inspect the building. The Lady Mayoress presented to Her Majesty a copy of the "Life of Edith Cavell," by Mr. Herbert Leeds. The inspection appeared to give the Royal visitors satisfaction and pleasure, and before leaving Her Majesty addressed the nurses, sympathising with their work, commending them for their diligence and the busy time they continuously enjoyed, and expressing confidence that they were doing a really splendid work.

### At Norfolk and Norwich Hospital.

Queen Alexandra then proceeded to the King Edward VII. Memorial Ward of the Norfolk and Norwich Hospital, where she was received by Sir Eustace Gurney, the chairman of the Board, and where the matron, Miss Cann, the secretary, Mr. Frank Inch, and many others were presented to her. In the hospital grounds were the sisters, nurses, and other members of the staff, together with those soldier patients who were able to leave their wards. Queen Alexandra was familiar with the building, its foundation-stone having been laid by the late beloved King Edward VII. in 1909. Queen Alexandra spent some time conversing with the patients, and was specially interested in the

### Queen Alexandra Visits Norfolk's Capital—(*continued*).

case of Private Thornton, an Irish Canadian, who had been wounded at Arras and had been admitted to the hospital in September last. He went into battle carrying on him a pocket Bible. The bullet which struck him passed right through it and caused the wound which made him a patient. The Queen was greatly interested in this book, which she closely examined, after which she congratulated Private Thornton upon his escape.

In one of the military wards the Queen noticed the presence of a blind pianist, Mr. Palmer, who voluntarily visits the hospital daily and cheers and amuses the patients with his music. The Queen, whose devotion to music is well known, with ready and gracious apprehension asked Palmer to play to her, and his gratification can be imagined. Her Majesty expressed her appreciation of the kind action and generous services, and the enjoyment which he had given to her, which she had had the privilege of sharing with the wounded warriors. Before leaving Her Majesty made a gracious little speech to the sisters and nurses, in the course of which she expressed the great interest she had always taken in their profession. She assured them of her sympathy in their work, and her gratitude for and pride in our nurses' courage and great services which had been splendid throughout the war.

Indefatigable as ever, Her Majesty, Queen Alexandra, next visited the Red Cross Hospital at the Bishop's Palace, and on leaving for Sandringham large crowds watched Her Majesty's progress through the streets and gave her a most cordial welcome. The crowd of people present at the unveiling, which hundreds of wounded soldiers, squads of nurses, and several ambulance units had the joy of attending, once more proved the depth of feeling and affection which Queen Alexandra inspires, and the uplifting encouragement which Her Majesty meets with, everywhere, on those many visits of sympathy and cheer to which so much of Her time and efforts are devoted.

## Figures and Tables

**Figure f1:**